# Quantitative peptide binding motifs for 19 human and mouse MHC class I molecules derived using positional scanning combinatorial peptide libraries

**DOI:** 10.1186/1745-7580-4-2

**Published:** 2008-01-25

**Authors:** John Sidney, Erika Assarsson, Carrie Moore, Sandy Ngo, Clemencia Pinilla, Alessandro Sette, Bjoern Peters

**Affiliations:** 1 La Jolla Institute for Allergy and Immunology, 9420 Athena Circle, La Jolla, CA 92037, USA; 2 Torrey Pines Institute for Molecular Studies, 3550 General Atomics Court, 2-129, San Diego, CA 92121, USA

## Abstract

**Background:**

It has been previously shown that combinatorial peptide libraries are a useful tool to characterize the binding specificity of class I MHC molecules. Compared to other methodologies, such as pool sequencing or measuring the affinities of individual peptides, utilizing positional scanning combinatorial libraries provides a baseline characterization of MHC molecular specificity that is cost effective, quantitative and unbiased.

**Results:**

Here, we present a large-scale application of this technology to 19 different human and mouse class I alleles. These include very well characterized alleles (e.g. HLA A*0201), alleles with little previous data available (e.g. HLA A*3201), and alleles with conflicting previous reports on specificity (e.g. HLA A*3001). For all alleles, the positional scanning combinatorial libraries were able to elucidate distinct binding patterns defined with a uniform approach, which we make available here. We introduce a heuristic method to translate this data into classical definitions of main and secondary anchor positions and their preferred residues. Finally, we validate that these matrices can be used to identify candidate MHC binding peptides and T cell epitopes in the vaccinia virus and influenza virus systems, respectively.

**Conclusion:**

These data confirm, on a large scale, including 15 human and 4 mouse class I alleles, the efficacy of the positional scanning combinatorial library approach for describing MHC class I binding specificity and identifying high affinity binding peptides. These libraries were shown to be useful for identifying specific primary and secondary anchor positions, and thereby simpler motifs, analogous to those described by other approaches. The present study also provides matrices useful for predicting high affinity binders for several alleles for which detailed quantitative descriptions of binding specificity were previously unavailable, including A*3001, A*3201, B*0801, B*1501 and B*1503.

## Background

T cells recognize a complex formed between a major histocompatibility complex (MHC) molecule and an antigenic peptide, or epitope. The identification of T cell epitopes is crucial to facilitate the study of the correlates of immunity. Different MHC molecules are associated with different peptide binding specificities, usually referred to as MHC peptide binding motifs. A large body of literature relates to the definition of MHC binding motifs for class I molecules of several different species, including humans, mice, chimpanzees and macaques (see, e.g., [1], for review). In general, class I MHC molecules recognize peptides of 9 to 10 residues in length and carrying residues with similar physiochemical specificity at main anchor positions. Typically, the main anchors are found in position 2 and at the C-terminus of the peptide ligand, although other anchor arrangements have been described for several alleles.

A variety of different methods are available to define MHC peptide binding motifs, each associated with its own advantages and disadvantages. The most common methods involve the pool sequencing of naturally presented MHC ligands or the evaluation of the binding capacity of individual peptide libraries. The pool sequencing approach is based on the bulk sequencing of peptides naturally bound to MHC following their elution with acidic buffers from the MHC peptide binding site. This is a remarkably simple and effective method, and has been applied with success in dozens of instances [1]. It immediately and reliably identifies the most dominant binding requirements of an MHC molecule. An additional unique advantage of this approach is the fact that it is based on the characterization of physiologically processed ligands. Disadvantages associated with this method are that it is only semi-quantitative, and typically identifies only the most canonical (stringent) motifs. This can be a drawback in terms of utilizing this method for epitope predictions, since it has been shown that many dominant epitopes do not carry canonical pool sequencing defined motifs. For example, the prototypical dominant human leukocyte antigen (HLA) A*0201 restricted influenza matrix 58–66 epitope (sequence GILGFVFTL) [2,3] does not contain the main anchor pattern associated with the HLA-A*0201 pool sequencing motif, which specifies the presence of L or M in position 2. Indeed, in a recent study we observed that 57% (8/14) of the HLA-A*0201 restricted vaccinia-derived epitopes identified did not conform with the A*0201 motif derived by pool sequencing analysis [4].

The most common alternative method for defining motifs is based on establishing quantitative MHC binding assays *in vitro*, and then testing series of individual peptides. These peptides are either single substitution analogs of high affinity binding epitopes or ligands, or large libraries of unrelated peptides. This method allows detailed probing of the relative role and chemical specificity of each position along the peptide sequence. A concern over this method when relying solely on single substitution analogs relates to the fact that it might reflect a binding mode specific to the particular parent peptide utilized as "wild type", although in practice the specificity patterns identified by single substitution analysis typically correspond well with those identified by other methodologies [5-19]. The same binding assay approach can be used to test large libraries of unrelated peptides (typically 100 or more) of a given size, and all carrying acceptable main anchor residues. As each peptide represents a unique sequence, this approach overcomes the concern associated with the single substitution approach that any pattern identified is dependent on the context of the specific "wild type" ligand.

Affinity data from individual peptides can be analyzed with different computational approaches to derive quantitative motifs that elucidate both primary and secondary influences on binding capacity with great detail (see, e.g., [1,9,20-38]). Predictions based on this type of data can give very accurate quantitative approximations of peptide binding, and can discriminate between candidate ligands bearing the same main anchor motifs. The most significant drawback of this approach is that it is dependent upon the availability of panels of several hundreds of allele specific peptides. As a result, this approach can be relatively labor intensive and expensive. Also, the selection of peptide sequences can introduce biases into the training data, for example by over or under representing residues at specific sequence positions.

An alternative approach to characterize the binding specificity of MHC molecules is based on the use of positional scanning combinatorial peptide libraries. Such libraries consists of combinatorial mixtures of large numbers of different peptides all sharing a single residue at a certain position. Measuring the affinity of such a library effectively evaluates the average influence of the shared residue on binding in a diverse set of surrounding sequences. Thus, an estimate of the binding contribution of all 20 residues in a 9-mer peptide can be derived by measuring the affinity of a set of 180 mixtures. This approach has been utilized successfully to determine specificity for several different applications, including analyses of the specificities associated with T cell receptor (TCR) recognition [39], proteosomal cleavage [40], and transporter associated with antigen processing (TAP) transport [41], as well as the identification of T cell epitopes [42,43]. Their efficacy in characterizing MHC binding specificity was first explored in several studies starting over a decade ago [32,44-46]. Matrices derived from analysis of combinatorial libraries have been found to perform well in the prediction of peptides with high MHC binding affinity [32,47,48]. Buus, in his visionary review of MHC studies, proposed the systematic use of combinatorial libraries a "Human MHC Project", directed at a complete mapping of human immune reactivities [49,50].

Like the single substitution or peptide library approaches, data generated from positional scanning combinatorial library studies provide quantitative motifs. The unique advantage of using positional scanning combinatorial libraries is that they can be re-used for every allele, representing potentially very significant cost savings. Retesting the same probes for each allele also removes the risk of introducing bias into the set of tested ligands. These advantages have led us to systematically apply the use of combinatorial libraries to a set of 19 class I MHC alleles. In this large scale evaluation, we test if this approach works uniformly across different alleles. We compare its prediction performance to that of bioinformatics machine learning algorithms. We also developed a heuristic approach to convert the combinatorial library affinity data into a classical representation of primary and secondary anchor positions, which makes them directly comparable to those obtained in pool sequencing. Finally, we test the ability of these matrices in practical applications to identify MHC binding peptides and T cell epitopes.

## Methods

### Positional scanning combinatorial libraries and peptide synthesis

The combinatorial library was synthesized as previously described [51]. Each pool in the library contains 9-mer peptides with one fixed residue at a single position. With each of the 20 naturally occurring residues represented at each position along the 9-mer backbone, the entire library consisted of 180 peptide mixtures.

Peptides utilized in screening studies were synthesized as described elsewhere [16], or purchased as crude material from Mimotopes (Minneapolis, MN/Clayton, Victoria, Australia), Pepscan Systems B.V. (Lelystad, Netherland) or A and A Labs (San Diego, CA). Peptides synthesized for use as radiolabeled ligands were synthesized by A and A Labs and purified to >95% homogeneity by reverse phase HPLC. Purity of these peptides was determined using analytical reverse-phase HPLC and amino acid analysis, sequencing, and/or mass spectrometry. Peptides were radiolabeled with the chloramine T method [52]. Lyophilized peptides were re-suspended at 4–20 mg/ml in 100% DMSO, then diluted to required concentrations in PBS +0.05% (v/v) nonidet P40 (Fluka Biochemika, Buchs, Switzerland).

### MHC purification and peptide binding assays

MHC purification and quantitative binding assays based on the inhibition of binding of a high affinity radiolabeled ligand were performed essentially as described elsewhere [18,52]. HLA A*0201, A*6802, B*0702, B*0801, B*2705, B*3501, B*5101, B*5301, and B*5401 molecules were purified from EBV transformed homozygous B cell lines, as previously described [15,16,18,52-55]. For A*3201, B*1501, B*5801 and B*5802, the WT47, SPACH, AP and 35841 cell lines were utilized, respectively. A*3001 molecules were obtained from the RSH cell line, or kindly provided by Dr. Soren Buus. B*1503 molecules were purchased from Pure Protein L.L.D. (Oklahoma City, OK), or kindly provided by Dr. Soren Buus. All HLA cell lines are from the IHWG cell bank (Fred Hutchinson Cancer Research Center). Mouse class I molecules were purified from P815 (H-2 D^d ^and K^d^), CH27 (H-2 K^k^), or EL-4 (H-2 D^b^) lines, as previously described [10,52].

For the B*1501, B*1503, A*3201 and A*3001 assays, the artificial sequences AQIDNYNKF (peptide 3128.0001), YQAVVPLVY (peptide 3054.0065), RILHNFAYSL (peptide 1454.42) and KTKDYVNGL (peptide 1428.02) were utilized as the radiolabeled probes, respectively. Radiolabeled ligands for all other assays were as previously described [15,16,18,52-55]. In competition assays, each mixture or individual peptide was tested in 3 or more independent experiments for its capacity to inhibit the binding of the radiolabeled peptide. The concentration of peptide yielding 50% inhibition of the binding of the radiolabeled peptide was calculated. Under the conditions utilized, where [label] < [MHC] and IC50 ≥ [MHC], the measured IC50 values are reasonable approximations of K_D_.

### Bioinformatic analysis

IC50 nM values for each mixture were standardized as a ratio to the geometric mean IC50 nM value of the entire set of 180 mixtures, and then normalized at each position as previously described [17,18] so that the value associated with the optimal value at each position corresponds to 1. For each position, an average (geometric) relative binding affinity (ARB) was calculated, and then the ratio of the ARB for the entire library to the ARB for each position was derived. We have denominated this ratio, which describes the factor by which the normalized geometric average binding affinity associated with all 20 residues at a specified position differs from that of the average affinity of the entire library, as the specificity factor (SF). As calculated, positions with the highest specificity will have the highest SF value. Primary anchor positions were then defined as those associated with an SF > 2.4. This criterion identifies positions where the majority of residues are associated with significant decreases in binding capacity. Secondary anchors were identified based on the standard deviation of residue specific values at each position.

To identify predicted binders, all possible 9-mer peptides in vaccinia WR sequences were scored using the matrix values, where the final score for each peptide represents the product of the matrix value for the corresponding residue at each position. Algorithms derived by combining positional scanning combinatorial library and individual peptide data sets were generated using the stabilized matrix method (SMM) approach, as previously described [56].

### Characteristics of the Study Population

Healthy males and females between 25 and 49 years of age were used in this study. Exclusion criteria were body weight of <45.4 kg and/or established pregnancy. Institutional Review Board approval and appropriate consent were obtained.

### Peripheral Blood Mononuclear Cell (PBMC) Isolation and HLA Typing

PBMCs were isolated from heparinized blood by gradient centrifugation with a Histopaque-1077 (catalogue no. H8889, Sigma) [57], and the cells were cryopreserved in liquid nitrogen in 10% DMSO/FBS. Each donor's PBMCs were typed for HLA-A and -B by high-resolution PCR (Atria Genetics, San Francisco, CA).

### Ex Vivo Primary ELISPOT Assay

Peptides were synthesized, and divided into groups according to their predicted HLA-A and HLA-B-restriction. PBMCs from individuals with the corresponding haplotype were incubated at 2 × 10^5 ^per well in the presence of individual peptides at 10 μg/ml, or a control pool with 24 peptides derived from commonly encountered pathogens (EBV, CMV, and influenza A virus) [58,59]. The ELISPOT assays were performed as described previously [60]. Responses against DMSO alone were subtracted from the experimental values. To assess statistical significance, a one-tailed Student *t *test was performed in which the triplicate values of each condition were compared with those of the negative controls. The criteria for positivity in a single experiment was set to ≥ 20 net spot-forming cells (SFCs)/10^6^, a stimulation index (SI) ≥ 2.0, and p ≤ 0.05. Each experiment was performed twice. Epitopes were defined as peptides giving a positive response in 2/2 experiments using PBMC from a single donor.

## Results

### Evaluation of the positional scanning combinatorial library approach for predicting HLA A*0201 binding peptides

Previous studies in other laboratories have demonstrated that the combinatorial approach performs well in predicting binders to several murine MHC class I molecules [32,46,47]. To verify that the same holds for human MHC molecules, we initially used the positional scanning combinatorial library with the best characterized human allele HLA A*0201, for which detailed primary and secondary anchor motifs have been described (see, e.g., [3,9,16,34,61,62]). Also, several different predictive methods for this allele are widely available, and have been rigorously tested and compared (see, e.g., [36,47]).

Previously, we compared the efficacy of several prediction approaches for A*0201 [47]. In that analysis, 3 algorithms hosted by the Immune Epitope Database (IEDB) [63-65] were evaluated in cross-validation, and 16 other publicly available algorithms were evaluated directly by scoring a library of over 3000 peptides whose capacity to bind A*0201 was known. The performance of each method was then evaluated using receiver operator curves (ROC), and calculating the area under the curve (AUC). The performance of the 16 directly evaluated algorithms, measured by AUC, ranged from a best of 0.935 to 0.788 (see [47] and Table 1). Overall, the average performance was 0.864, with a median score of 0.871.

**Table 1:** Performance of several methods for predicting A*0201 binders.

**Method**	**AUC**	**Rank**
arbmatrixa	0.935	1
netmhcanna	0.934	2
hla_a2_smm	0.922	3
bimas	0.920	4
mapppB	0.920	4
mhcpathwaya	0.915	6
multipredann	0.883	7
mapppS	0.871	8
syfpeithi	0.871	8
rankpep	0.836	10
hlaligand	0.816	11
mhcpred	0.814	12
svmhc	0.814	12
multipredhmm	0.796	14
pepdist	0.789	15
predep	0.788	16

Average	0.864	
Median	0.871	

Combinatorial library	0.909	

1. With the exception of the positional scanning combinatorial library, these data have been previously reported [47], and are shown here for reference purposes.

**Figure 1:**
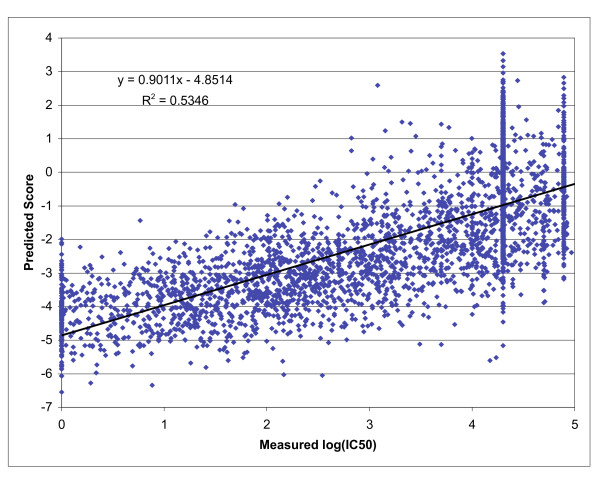
Positional scanning combinatorial library based predictions for HLA A*0201. Scatter plot depicting the relationship between the predicted score generated from the A*0201 matrix and measured IC50 nM values for 3089 9-mer peptides. Binding assays were performed as described in the materials and methods for peptides previously [47] utilized to compare various publicly available prediction tools. Peptides were scored using the matrix as described in the text.

To gauge the relative performance of the combinatorial approach, each of the 9-mer mixtures was tested for its capacity to inhibit the binding of a high affinity radiolabeled ligand to purified A*0201 molecules. The measured IC50 nM values for each mixture are shown in Additional file 1 [see Additional file 1]. IC50 nM values were then normalized as described in the Materials and Methods. The resulting A*0201 matrix (Table 2) was used to score the same 3089 9-mer peptides used above. Good agreement between predicted IC50 and measured IC50 was noted (r^2 ^= 0.53), and an AUC of 0.909 was measured (Table 1 and Figure 1). This performance is above both the average and median found for 10 of the 16 other algorithms available on the Internet. This performance is notable in that the combinatorial library utilizes only 180 data points as the training set. By contrast, the top performing ARB, ANN and SMM-based algorithms were developed using a training set with over 10-fold more data points.

**Table 2:** Positional scanning combinatorial library derived matrix describing 9-mer binding to HLA A*0201.

	**Position**								
	
**Residue**	**1**	**2**	**3**	**4**	**5**	**6**	**7**	**8**	**9**
A	0.114	0.002	0.431	0.895	0.377	0.715	0.334	0.259	0.105
C	0.032	0.002	0.222	0.201	0.758	0.435	0.260	0.178	0.005
D	0.008	0.002	0.465	0.915	0.114	0.211	0.149	0.161	0.003
E	0.015	0.030	0.037	0.647	0.193	0.838	0.536	0.492	0.038
F	1.000	0.004	0.630	0.359	0.322	0.364	0.696	0.784	0.084
G	0.089	0.009	0.098	0.537	0.270	0.152	0.194	0.358	0.008
H	0.005	0.002	0.270	0.168	0.184	0.207	0.345	0.164	0.002
I	0.121	0.057	0.537	0.321	0.300	0.843	1.000	0.082	0.327
K	0.114	0.012	0.016	0.372	0.182	0.088	0.041	0.199	0.002
L	0.054	1.000	0.568	0.661	0.263	0.555	0.346	0.357	0.181
M	0.251	0.186	1.000	0.291	1.000	0.363	0.353	0.264	0.075
N	0.067	0.006	0.159	0.823	0.329	0.725	0.385	0.177	0.003
P	0.003	0.003	0.056	0.792	0.109	0.328	0.957	1.000	0.003
Q	0.019	0.140	0.200	0.582	0.104	0.566	0.703	0.190	0.002
R	0.084	0.002	0.019	0.233	0.220	0.128	0.072	0.306	0.002
S	0.103	0.009	0.167	1.000	0.179	0.190	0.533	0.377	0.008
T	0.094	0.038	0.145	0.189	0.199	1.000	0.236	0.267	0.015
V	0.062	0.098	0.246	0.282	0.263	0.734	0.284	0.243	1.000
W	0.057	0.002	0.584	0.637	0.331	0.197	0.628	0.352	0.005
Y	0.107	0.008	0.875	0.303	0.235	0.150	0.946	0.609	0.002

Average	0.054	0.012	0.203	0.440	0.249	0.348	0.347	0.287	0.013
SD	3.9	6.6	3.3	1.8	1.8	2.1	2.3	1.8	7.4
SF	2.28	10.36	0.61	0.28	0.49	0.35	0.35	0.43	9.42

Library average: 0.123

While information of training set size is unavailable for the remaining algorithms, given the general availability of A*0201 binding data, it is reasonable to assume that these algorithms have utilized training sets of similar size. Furthermore, the training set for the combinatorial approach does not overlap with the test set, and is thus completely unbiased, unlike the case for most of the tools utilized in the comparison [47]. Taken together, the data presented in this section have provided further demonstration of the efficacy of using a positional scanning combinatorial library for identification of MHC class I binding peptides.

### Generation and validation of positional scanning combinatorial library matrices for additional human and murine class I alleles

Encouraged by the results obtained in the context of A*0201, we derived, and make herein available to the scientific community, combinatorial library matrices for an additional 14 HLA (A*3001, A*3201, A*6802, B*0702, B*0801, B*1501, B*1503, B*2705, B*3501, B*5101, B*5301, B*5401, B*5801 and B*5802) and 4 mouse (H-2 D^d^, K^d^, D^b ^and K^k^) class I molecules. The measured IC50 values are provided in Additional file 1 [see Additional file 1], and will also be submitted to the Immune Epitope Database (IEDB) for hosting at the IEDB Analysis resource [66]. The IC50 values for each mixture were normalized as described above. The resulting matrix values are tabulated in Additional file 2 [see Additional file 2]. For each allele, the matrices identified a reproducible, characteristic, binding pattern.

### Identification of primary and secondary anchor positions by the positional scanning combinatorial library approach

To compare the results of combinatorial matrices with those from pool sequencing and single residue substitutions, and in order to meaningfully summarize the rather large amount of data in each scoring matrix, it is desirable to describe MHC binding in terms of simple motifs. The first step in defining such a motif is identifying the peptide positions that have the strongest influence on binding.

As before, A*0201 was first utilized as a model system. A*0201 binds peptides utilizing the peptide residues in position 2 and at the C-terminus as main anchors. At both main anchor positions hydrophobic or aliphatic residues are preferred or tolerated. Additional influences on binding capacity are contributed by residues at secondary positions, most prominently positions 1, 3, and 7, where both positive and deleterious influences can be noted [9].

To derive an objective criteria useful for identifying primary anchor positions, we reasoned that positions associated with the highest specificity would be associated with the lowest average affinity, as the majority of residues would not be tolerated. Accordingly, we first calculated the average relative binding affinity (ARB) for each position, representing the geometric mean of the values at each position (see Table 2), normalized to the average for the entire library. We have denominated this ratio as the specificity factor (SF). Analysis of the A*0201 data revealed that a SF value of ≥ 2.4 could demarcate only position 2 and the C-terminus as the main anchor positions (Table 3). To test the general applicability of the SF method for identifying primary anchors, we generated SF values for each of the 18 other alleles for which we have derived combinatorial matrices (Table 3). For 13 of the 18 additional alleles examined (72%), a SF > 2.4 identified all main anchor positions identified by pool sequencing or other motif analyses. Overall, 33/38 (87%) anchor positions identified previously were identified using this criteria. Notable exceptions were A*3001 and B*0801. Using a higher or lower threshold resulted in lower correspondence with previously described motifs.

**Table 3:** Specificity factors derived from positional scanning combinatorial library matrices identify primary anchor positions.

		**SF**^1^								
		
**System**	**Allele**	**P1**	**P2**	**P3**	**P4**	**P5**	**P6**	**P7**	**P8**	**P9**
HLA	A*0201	2.28	**10.36**	0.61	0.28	0.49	0.35	0.35	0.43	**9.42**
	A*3001	1.41	0.72	**7.54**	0.27	0.52	0.65	0.96	0.45	**3.31**
	A*3201	0.89	**2.85**	0.57	0.64	0.81	0.44	1.06	0.50	**5.69**
	A*6802	1.32	**4.59**	1.13	0.62	0.48	0.38	0.41	0.84	**3.75**
	B*0702	0.59	**13.70**	1.43	0.48	0.42	0.40	0.30	1.16	**3.06**
	B*0801	0.85	1.05	1.10	0.31	**2.53**	**2.78**	0.35	0.54	**2.44**
	B*1501	0.70	2.11	0.72	0.53	0.37	0.71	0.49	1.07	**12.95**
	B*1503	0.73	**3.22**	0.92	0.34	0.48	0.43	1.08	0.74	**8.17**
	B*2705	0.77	**36.73**	0.77	0.50	0.85	0.79	0.41	0.39	0.83
	B*3501	0.94	**3.55**	0.75	0.86	0.41	0.61	0.54	0.71	**4.79**
	B*5101	1.34	**2.57**	0.59	0.29	0.46	0.59	0.37	0.44	**39.03**
	B*5301	2.07	**3.68**	1.29	0.41	0.59	0.36	0.57	0.36	**5.80**
	B*5401	1.02	**9.12**	2.21	0.28	0.32	0.32	0.31	0.67	**8.30**
	B*5801	0.81	**4.49**	0.71	0.43	0.68	0.45	1.02	0.64	**4.54**
	B*5802	1.47	1.79	0.74	0.37	0.57	0.57	2.08	0.50	**4.10**

H-2	Db	1.18	1.07	1.39	0.35	**3.61**	0.28	0.42	0.79	**4.93**
	Dd	0.37	**3.24**	**17.14**	0.21	0.59	0.22	2.29	0.20	**3.89**
	Kd	0.31	**52.55**	0.61	0.51	0.73	0.38	0.75	0.23	**4.14**
	Kk	0.36	**8.69**	0.56	0.35	1.06	0.32	1.20	0.49	**8.13**

1. SF > 2.4, identifying primary anchor positions, are highlighted by bold font.

**Figure 2:**
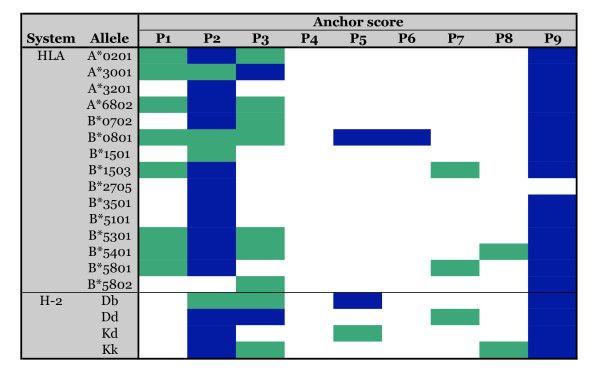
**Primary and secondary anchor positions for 19 HLA and H-2 class I alleles defined using positional scanning combinatorial peptide library matrices**. Maps of primary and secondary anchor positions as defined using the combinatorial library data. Primary anchors (blue shading) were identified using specificity factors (SF), as secondary anchor positions (green shading) were determined on the basis of standard deviation (SD), as described in the text.

To define secondary anchor positions a different approach was utilized. We reasoned that positions associated with the highest standard deviation (SD) between residues would correspond to those most affected positively or negatively by secondary binding effects. Again analyzing the A*0201 data, it was found that a SD > 3 could successfully identify positions previously described as secondary anchors (Figure 2). Using a higher or lower threshold resulted in lower correspondence with previously described motifs. Applying this criterion to the other alleles, one or more dominant secondary anchor positions could be identified for most alleles (Figure 2). In the majority of these cases, the dominant secondary anchors were found to be in positions 1, 3 and 7. This pattern of secondary interactions is largely in agreement with a previous analysis [67].

### Validation of the positional scanning combinatorial library approach: identifying primary anchor preferences

We next examined how well the preference patterns at the identified main anchor position agreed with those identified by pool sequencing or peptide library methods. Again starting with the well characterized A*0201 allele, we found that residues with relative affinities within about 10-fold of the optimal residue correspond to those previously described as preferred at the position 2 and C-terminal main anchors (Tables 2 and 4). More specifically, at position 2, using a 10-fold threshold identified L, M and Q as the most preferred residues. By contrast, the pool sequencing approach identified L and M. Similarly, at the C-terminus, V, I, L and A were identified as the preferred residues by the combinatorial libraries, compared to V and L from pool sequencing. In this respect, the preferences identified by the 10-fold criteria for combinatorial libraries are about mid-way between the more stringent motif defined by pool sequencing analyses, and the extended motif identified by peptide screening approaches [16].

**Table 4:** Comparison of main anchor motifs identified using positional scanning combinatorial libraries with those using other approaches.

		**Published motif**					**Combinatorial library motif**				
			
**System**	**Allele**	**P2**	**P3**	**P5**	**P6**	**P9**	**P2**	**P3**	**P5**	**P6**	**P9**
HLA	A*0201	LM [IVATQ]				VL [MIAT]	L [MQ]				VI [LA]
	A*3001	YF				L		RK			KA [LVIY]
	A*3201	[MLITVQS]				[WIFYHT]	TMIQLVS [A]				FIYLW
	A*6802	[LMIVATQS]				[LMIVAT]	VTS [IALMP]				VALI
	B*0702	P				L [FWYIVMA]	P [VA]				LFAVI [M]
	B*0801		RK	RK		LIVM			RHKF	F [RH]	LFMVIA [E]
	B*1501	QL				FY					FY [M]
	B*1503	QK				FY	QMK [LHASE]				F [MY]
	B*2705	R				KRLYANFMIH	R				
	B*3501	P				YFMLI [WVA]	PA				FYM [A]
	B*5101	APG				VI [FWYLMA]	PA [GQVS]				I [V]
	B*5301	P				WFL [YIVMA]	PA [IV]				FC [IW]
	B*5401	P				[FWYLIVMA]	A [P]				AV
	B*5801	AST				FW	STA [VG]				WFIY [MC]
	B*5802	ST			R	F					IFL [MW]

H-2	Db			N		LIVM			N [L]		IML [VF]
	Dd	G	P	RK		LFI	G	P			FLI [C]
	Kd	YF				LIVM	Y				ILV [M]
	Kk	E				LIVM	E [D]				IV [FL]

1. Indicates residues preferred in the indicated anchor positions, as reported in the published literature. Motifs are as identified by pool sequencing motifs. Residues in brackets are additional residues identified on the basis of peptide binding studies.

2. Indicates residues preferred in the indicated anchor positions, as identified by the combinatorial library approach. Motifs are as identified using an ARB > 0.2 threshold. Residues in brackets are additional residues identified when the threshold is lowered to 0.1.

The same criteria were then applied to the set of 18 additional alleles. Again, the patterns identified by the combinatorial libraries largely followed those previously described (Table 4). As was the case with A*0201, the 10-fold criteria applied to the combinatorial library data tended to identify a broader motif than identified by pool sequencing. However, when a more stringent threshold (e.g., 5-fold) is utilized, a narrower motif very similar to that described by pool sequencing is identified.

This analysis revealed several unexpected designations. The identification of position 3 as a main anchor for A*3001 binding, instead of position 2, is in disagreement with the published literature, but was not entirely unexpected based on analyses using single amino acid substitution peptides (Sidney and Sette, unpublished observations). The preference in position 2, identified as a dominant secondary anchor here, appears to be more towards small residues (V, T, and A) rather than aromatics, as indicated by pool sequencing, although these latter residues are still well tolerated. The preference at position 3 was found to be for basic residues. Pool sequencing had suggested a preference for hydrophobic residues at the C-terminus. While the combinatorial library generated motif is not in disagreement with this general specificity, the identification of an A3-supertype like preference for K was unexpected. However, subsequent MHC peptide binding studies by others (Harndahl and Buus et al, IEDB submission 1000945, [63]) and us (Sidney and Sette, unpublished observations) have confirmed this preference. Positions 1 and 2 were found to be dominant secondary anchors. This observation, in consideration with the discrepancy identified between the pool sequencing and positional scanning combinatorial libraries, suggests that A*3001 may be able to bind peptides using multiple different anchor arrangements.

In other cases, specifically B*1501, B*5802 and B*2705, no clear anchors were defined at either the N- or C-terminal end, where a more diffuse chemical specificity is apparent. A similar failure to identify dominant signals at more than one anchor residue has also been noted for several alleles when using pool sequencing methods (see e.g., [68]).

A detailed motif for A*3201 has not, to our knowledge, been previously made available. It has been suggested that this allele would be a member of the A1-supertype, and the motif identified herein is congruent with that association. However, peptide binding studies have not yet been able to confirm that this allele shares significant repertoire overlap with other alleles of this supertype.

### Application of positional scanning combinatorial libraries: Predicting MHC binding peptides

**Figure 3:**
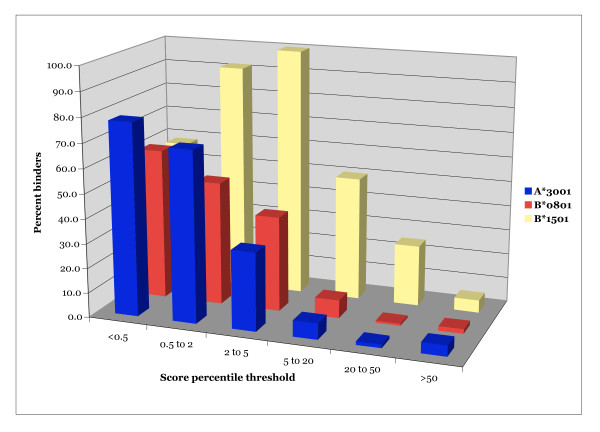
**Efficacy of positional scanning combinatorial library based predictions for 3 HLA class I alleles**. The percent of peptides scoring within a specified percentile range that bind A*3001, A*3201 or B*1501. Peptides were scored using the corresponding combinatorial library matrix. Peptides were then assigned a percentile score indexed to the percentile associated with 9-mer peptides derived from vaccinia with the same matrix score. About 60,000 9-mers derived from the vaccinia WR sequence were scored to develop the indices.

The performance of combinatorial library matrices was further evaluated with 5 selected alleles (A*3001, A*3201, B*0801, B*1501 and B*1503). Each of these alleles is relatively common in the human population, but large sets of high affinity binding peptides are not, to our knowledge, currently available. Using these matrices, all 9-mer sequences from vaccinia Western Reserve (WR) strain were scored utilizing the product of the matrix value for the corresponding residue at each position. For each allele, the top 300 scoring 9-mers peptides, corresponding to approximately the top 0.5%, were synthesized and tested for binding. The binding data are summarized in Additional file 3 [see Additional file 3]. It was found that on average 68% of the peptides selected bound their corresponding allele with an affinity of 100 nM or better, with a minimum of 58% in the case of B*1501, and a maximum of 78% in the case of A*3001. By comparison, in the cases of A*3001, B*0801 and B*1501, where binding data was available for sets of peptides with poorer scores, it was found that peptides with scores equivalent to those in the lower 50% range were only rarely binders, with rates of binding in the 1 to 5% range (Figure 3).

Taken together, these data further validate the use of combinatorial libraries as a basis for predictive algorithms. Also, the present analysis has provided sets of high affinity binders derived from vaccinia WR for 5 relatively common HLA class I alleles.

### Application of positional scanning combinatorial libraries: Predicting T cell epitope candidates

We initially wanted to test the sets of high affinity peptides identified from vaccinia virus in DryVax immunized donors, similar to a previous investigation with donors carrying HLA alleles from common supertypes [69]. However, at the time of this study, we were unable to enroll a large enough number of newly vaccinated donors with the desired matching HLA alleles. We instead decided to validate the ability of combinatorial libraries to aid in the identification of T cell epitopes from influenza recognized in human donors for which we could enroll multiple donors with the HLA alleles A*3001, A*3201, and B*1501.

To make optimal use of both the combinatorial library data and the individual peptide binding data for these alleles, we utilized the SMM (stabilized matrix method) approach [56], which can combine these data to compute second generation matrices. These second generation matrices have been found to perform better than predictions based on either approach alone. All 9-mer peptides present in a representative set of Influenza A H1N1 and H3N2 strains were scored using the second generation matrices for A*3001, A*3201, and B*1501 as shown in Additional file 4 [see Additional file 4], and for each allele the top 100 scoring peptides were synthesized.

The predicted high-affinity-binding peptides were tested for their ability to elicit T cell responses from human donors with matching HLA. PBMCs from donors were isolated from leukopherisis or general blood donation volunteers, and HLA-typed by high-resolution PCR. In total, 13 healthy donors of 25–49 years of age were included in the study, including 3 donors for A*3001 and A*3201, and 8 for B*1501. Cryopreserved PBMCs were assayed with individual peptides from the set(s) corresponding to the donor's haplotype, and the reactivity was determined using IFNγ ELISPOT assays. Positive epitopes were defined as described in the Methods.

From these experiments, epitopes were successfully identified for each allele (Table 5). Specifically, 2, 1, and 13 epitopes were identified in patients typed as A*3001, A*3201 and B*1501, respectively. However, no peptide was recognized in more than one donor. To the best of our knowledge, each of these represent novel epitopes, and together are the first set of influenza virus derived epitopes based on predictions for A*3001, A*3201 and B*1501.

**Table 5:** Influenza epitopes.

**Putative restriction**	**Peptide**	**Sequence**	**Average SFC/10^6**
A*3001	NP.358	GTKVIPRGK	32
A*3001	PB2.219	KTRFLPVAG	45
A*3201	NA.369	KLRSGYETF	57
B*1501	HA.91	LLPARSWSY	67
B*1501	HA.113	RSKAFSNCY	25
B*1501	HA.219	YVSVVSSHY	83
B*1501	HA.361	GMIDGWYGF	104
B*1501	M1.164	QMVTTTNPL	73
B*1501	NA.32	LVTTVTLHF	59
B*1501	NA.363	KSNSSRRGF	110
B*1501	NA.371	KSRSGYETF	137
B*1501	NP.404	GQISVQPTF	59
B*1501	NP.404	GQISTQPTF	42
B*1501	NS1.134	MLKANFSVI	108
B*1501	PB1.623	RLCNPLNPF	91
B*1501	PB2.198	LQNCKISPL	59

## Discussion

Because peptide binding to MHC is a requirement to elicit a T cell response, algorithm-based approaches predicting peptide binding are often utilized as a first screen to identify epitopes derived from large pathogens. In the present study, we have utilized 9-mer positional scanning combinatorial libraries to characterize the peptide binding specificities of several mouse and human class I alleles. When the corresponding positional scanning combinatorial library data were utilized to generate matrices for the prediction of binders derived from vaccinia, it was found that in all cases examined between 58 and 78% of the top 0.5% scoring peptides were high affinity binders, depending on the specific allele considered. The biological relevance of quantitative motifs derived from combinatorial library analyses were validated by identifying several epitopes derived from influenza A virus that were recognized by PBMCs from human donors. This study therefore provides a set of 19 uniformly generated matrices that can be directly applied to predict MHC peptide binding and T cell epitope candidates.

An implicit feature of the approach is that it provides a detailed quantitative motif for each MHC specificity examined. However, it is often useful to summarize MHC binding specificity in the more simple terms of primary anchor motifs. This "minimalist" approach dates back to the earliest studies of MHC binding, where specificities were defined using pool sequencing or single amino acid substitution analyses. These methods were very good at characterizing the most prominent features of allele specific motifs, and the resulting motifs have generally formed the syntax with which MHC binding is described. To extend the utility of the combinatorial approach, we have developed a heuristic approach to translate the matrix data generated by the combinatorial libraries into the more simple motifs that are the idiom of MHC studies. In the majority of cases, generalizable parameters could be defined that allowed the identification of main and secondary anchor positions congruent with those defined by other approaches.

The majority of HLA class I molecules whose binding specificity have been described by crystal structure, pool sequencing or peptide binding studies, the main anchor interactions of the peptide almost invariably involve the residues at position 2 and the C-terminus of the peptide. This pattern also appears to be true for most macaque and chimpanzee class I alleles studied to date. As evidenced by the cases of A*3001 and B*0801, the combinatorial library analysis suggests that the paradigm of position 2/C-terminus anchor spacing for MHC peptide binding is not always true. This has been reported previously in the case of B*0801 [70], where positions 3 and 5, in addition to the C-terminus, have been identified as primary anchors. Although this exact pattern was not duplicated by the combinatorial analysis, the present data do confirm the importance of positively charged residues in the middle of the peptide for conferring high affinity binding capacity. The ability to pick up unexpected binding patterns of MHC alleles is one of the key advantages of the combinatorial libraries, which have no prior expectations on which positions are likely to be important for MHC:peptide interactions.

In our previous HLA supertype classification study [71], B*0801 was considered an outlier on the basis of it's somewhat unique, for HLA, use of positions 3 and 5 as main anchor positions. This designation was also made by Lund [72] and Hertz [73]. Others [74,75] have classified it with alleles we [71] and others [72,73] have assigned as members of the B7-supertype. In the present study, the combinatorial library analysis suggested that positions 5 and 6 are important (in addition to the C-terminus) for peptide binding. As such, the present analysis does not provide sufficient evidence to suggest that B*0801 should be assigned to a specific supertype, which are largely defined by position 2 (and C-terminus) specificity. We can note that proline, the B7-supertype associated position 2 specificity, does appear to be well tolerated in position 2 by B*0801. Also, our own unpublished binding data suggests that there may be some cross-reactivity between B*0702 and B*0801. However, this potential cross-reactivity has not been examined in enough detail at this point to draw any conclusions.

Previously, B*3501, B*5101, B*5301, and B*5401 were assigned to the B7-supertype [71-73], which describes a set of HLA alleles sharing a preference for proline as the position 2 main anchor. In the present study, the combinatorial library analysis confirmed this preference, but, surprisingly, also indicated that alanine was well tolerated by these alleles in position 2. This would suggest that overlap may exist in the repertoires of at least some B7-supertype alleles with alleles outside the B7-supertype, and in particular those associated with the B58- and or B62-supertypes. While the binding data we have to date suggest that the majority of instances of repertoire overlap for B7-supertype alleles will fall within the B7-supertype, and that proline is the most dominant preference in position 2, evidence for some cross-reactivity is also quite apparent. Indeed, a recent study [76] has found a high degree of cross-recognition of epitopes between alleles associated with different supertypes. Future studies will hopefully shed additional light on this issue.

In utilizing combinatorial libraries to characterize MHC specificity and identify binders, the approach we have implemented is computationally simple. We have largely utilized relative binding values for each residue/position coordinate. To predict binders, we have assumed the independent binding of peptide side chains, and represented the predicted binding propensity as a product of each coordinate. There are other ways to process the raw data for the purpose of generating prediction matrices, or to define anchor positions. To facilitate further investigation of prediction approaches by the bioinformatic community, we have here provided both the raw and processed data for over a dozen different HLA, and 4 H-2, class I alleles. We believe that this data will be of value to the community for the prediction of binders and epitopes, at least for several alleles not previously characterized in detail.

We compared the prediction performance of the combinatorial library with a set of 16 bioinformatic approaches for the best characterized human MHC allele, HLA A*0201. While several algorithms outperformed the combinatorial library, this has to be taken into perspective, as these algorithms are based on up to ten times more training data. Even more surprising, the combinatorial library nevertheless proved highly competitive, with a better prediction quality than 10 out of 16 algorithms. Taken together, the combinatorial libraries minimally provide a very solid baseline characterization of MHC binding specificity, which can be generated both quickly and with cost effectiveness.

Using combinatorial library based matrices to identify sets of candidate peptides, epitopes were successfully identified in patients typed as A*3001, A*3201 and B*1501. Notably, however, no peptide was recognized in more than one donor. This diversity of responses is similar to what was noted previously for mapping T cell responses to vaccinia-derived peptides in human donors. Because of the small number of A*3001 and A*3201 donors tested, it is possible we have under estimated the number of positive responses. Other factors may also be responsible for the lower response rates observed. The donor pool utilized represents an outbred population, and almost all of the donors were heterozygous at both the A and B loci. Thus, the diverse donor responses may reflect the different influences of other MHC alleles in shaping the overall T cell repertoire. Similarly, that dominant epitopes recognized in multiple donors were not identified may be due to the fact that the set of donors is representative of diverse histories of exposure to different viral strains.

The epitope identification aspect of the study was not pursued to the level and detail of our previous studies (e.g., [69]). Several factors are responsible for this, including the fact that while the alleles studied are not rare, neither are they prevalent, making it resource intensive to identify a sufficient number of additional donors. As a result, the identified peptides represent potential leads of a preliminary nature. At the same time, the data does help demonstrate that the matrices derived in the study are useful for epitope identification, even if the epitope identification study was not ideal. Furthermore, to the best of our knowledge, each of the epitopes identified in the present study represent novel epitopes, and together are the first set of influenza virus derived epitopes based on predictions for A*3001, A*3201 and B*1501.

## Conclusion

The present study has extended observations from previous studies [32,44,46,48,49] showing the usefulness of positional scanning combinatorial libraries for identifying MHC class I binding peptides. Herein we have made available combinatorial library based matrices for 19 class I alleles of human and mouse origin, including several that have not previously been characterized in detail. These libraries have also been shown to be useful for identifying specific primary and secondary anchor positions, and thereby simpler motifs, analogous to those described by other approaches. For A*3001, A*3201, B*0801, B*1501 and B*1503, sets of vaccinia WR derived peptides that bind with high affinity have been identified. These peptides represent candidates for future studies towards the identification of epitopes derived from vaccinia, a virus of high interest for the development of viral vector based vaccines, in addition to its well-known use as a vaccine against smallpox. Finally, we have also identified several epitopes derived from influenza that are recognized in HLA A*3001, A*3201 and B*1501 donors.

## Competing interests

The author(s) declare that they have no competing interests.

## Authors' contributions

JS performed the MHC binding data analyses and drafted the manuscript. EA performed all cellular immunology assays and associated data analyses, and assisted preparing the manuscript. AS and BP participated in the conceptualization and design of the study, provided interpretation of the data, and assisted in the preparation of the manuscript. CM and SN performed all MHC binding assays and MHC purification. CP participated in conceptualization of the study, designed and provided the positional scanning combinatorial libraries, and helped to draft the manuscript. All authors read and approved the final manuscript.

## Supplementary Material

Additional file 1 Binding capacity of positional scanning combinatorial libraries for 19 HLA and H-2 class I alleles. This table lists the measured IC50 nM value of each mixture in the combinatorial library for 19 class I alleles. Binding assays were performed as described in the Methods section. Click here for file

Additional file 2 Normalized average relative binding affinities of positional scanning combinatorial libraries for 19 HLA and H-2 class I alleles. This table lists the normalized ARB values of each mixture in the positional scanning combinatorial library for 19 class I alleles. Binding data for each mixture were normalized as described in the materials and methods, such that the optimal mixture (residue) at each position corresponds to 1.0, and residues associated with lower affinities with correspondingly lower values. The normalized values for A*0201 are also shown in matrix format in Table 2. Click here for file

Additional file 3 Vaccinia WR-derived peptides that bind 5 common HLA alleles with high affinity. The vaccinia WR-derived peptides identified that bind A) A*3001, B) A*3201, C) B*0801, D) B*1501 or E) B*1503 with affinities of 500 nM, or better are shown. Candidate peptides were selected using the corresponding normalized combinatorial library matrix values [see Additional file 2], and tested for binding as described in the Methods. For each allele, a set of 300 candidate peptides was originally selected. Click here for file

Additional file 4 Second generation matrices for predicting binders to HLA A*3001, A*3201 and B*1501. Second generation prediction matrices were derived combining the positional scanning combinatorial library and peptide library data, using the stabilized matrix method (SMM) approach, as previously described [56]. Scores for individual peptides represent log predicted IC50 nM values, and are calculated as the sum of the corresponding position/residue values, plus the constant. Algorithms derived by combining positional scanning and individual peptide data sets were generated. Click here for file
